# Caffeine Caused a Widespread Increase of Resting Brain Entropy

**DOI:** 10.1038/s41598-018-21008-6

**Published:** 2018-02-09

**Authors:** Da Chang, Donghui Song, Jian Zhang, Yuanqi Shang, Qiu Ge, Ze Wang

**Affiliations:** 1 Center for Cognition and Brain Disorders, Department of Psychology, Hangzhou Normal University, Yuhang, Hangzhou China; 2 Department of Radiology, Lewis Katz School of Medicine, Temple University, Philadelphia, PA 19122 USA

## Abstract

Entropy is an important trait of brain function and high entropy indicates high information processing capacity. We recently demonstrated that brain entropy (BEN) is stable across time and differs between controls and patients with various brain disorders. The purpose of this study was to examine whether BEN is sensitive to pharmaceutical modulations with caffeine. Both cerebral blood flow (CBF) and resting fMRI were collected from sixty caffeine-naïve healthy subjects before and after taking a 200 mg caffeine pill. Our data showed that caffeine reduced CBF in the whole brain but increased BEN across the cerebral cortex with the highest increase in lateral prefrontal cortex, the default mode network (DMN), visual cortex, and motor network, consistent with the beneficial effects of caffeine (such as vigilance and attention) on these areas. BEN increase was correlated to CBF reduction only in several regions (−0.5 < r < −0.4), indicating a neuronal nature for most of the observed BEN alterations. In summary, we showed the first evidence of BEN alterations due to caffeine ingestion, suggesting BEN as a biomarker sensitive to pharmaceutical brain function modulations.

## Introduction

Human brain is a dynamic complex system with large-scale ongoing fluctuations. Understanding those dynamic features is essential to our understanding of functional anatomy–and the pathologies associated with neuropsychiatric conditions. The key aspect of these dynamics is their complexity with higher complexity indicating a greater capacity for processing both internal or external inputs, which might be a requirement for the brain to adapt to the dynamically changing environment, characterized by a high occurrence of unpredictable events. Complexity has been addressed in many ways since the advent of the whole brain imaging^1–6^. The two main approaches are based upon information theory–using measures like entropy and mutual information^1,4^ – and dynamical system theory to address emergent phenomena – like self organized criticality and metastability^5–7^.

The first studies of brain complexity (based on fMRI) used information theory^1,2^: Because complex patterns of activity in the brain are intermediate between a state of incoherence, with regionally specific dynamics and a state of global coherence, greatest complexity is found somewhere between high-dimensional, chaotic behaviour and low-dimensional, orderly behaviour. This means that complexity is high when small regions have (on average) relatively high entropy with respect to the entropy of the whole system^2^. This measure is equivalent to the (average) mutual information between all small regions and the rest of the system in question and was shown to be higher than chance in fMRI signals acquired from the brain^2^. In effect, this means that long range spatial correlations are greater than would be predicted by short-range correlations.

The overall picture of a complex regime for neuronal dynamics–that lies somewhere between a low entropy coherent regime (such as coma or slow wave sleep) and a high entropy chaotic regime–also emerges from simulations of neuronal dynamics. For example, Deco and Jirsa^6^ used simulations to show that realistic dynamics–on realistic anatomical connections–show a characteristic metastability, leading to critical behaviours. This is consistent with the literature on self organized criticality in neuronal populations (e.g.^5^). A crucial aspect of complex dynamics–and in particular criticality–is the emergence of long range temporal correlations. This complements the information theory based characterizations of complexity above–that focused on the emergence of long range spatial correlations.

The potential importance of long range temporal correlations is highlighted by the recent focus on slow fluctuations in fMRI timeseries and the intrinsic spatial modes that they define–for example the default mode^8^. However, measuring the temporal complexity of fMRI timeseries in a robust and assumption free manner is a nontrivial issue. We have recently proposed a method^9^ to map the whole brain temporal complexity using a nonparametric entropy metric, the Sample Entropy^10,11^. This measure is based upon the entropy of measured haemodynamic states that considers dependency over time using temporal embedding. In other words, this use of entropy reflects the statistical dependencies or order implicit in itinerant dynamics, expressed over extended periods of time.

Using the brain entropy (BEN) mapping tool, we have demonstrated that regional BEN can be reliably mapped in the normal brain using resting state fMRI (rsfMRI)^9^. Normal brain presents structurally and functionally meaningful BEN distribution patterns at rest^9^. In subsequent studies, we showed BEN alterations in different brain disorders such as multiple sclerosis^12^, chronic cigarette smoking^13^, and cocaine addiction^14^, in complimentary to BEN alterations in aging^15^, Schizophrenia^16^, and attention deficit hyperactivity disorder (ADHD)^17^ reported by other groups. Altogether, these studies suggest BEN measured with rsfMRI as a reliable index of regional temporal brain dynamics, which is sensitive to regional functional modulations such as task activation and disease conditions. An important question to be answered next is whether BEN is sensitive to pharmaceutical modulations.

The purpose of this study was to examine sensitivity of BEN to caffeine, the most widely consumed psychostimulant^18–20^. Caffeine is an antagonist to adenosine^20^, a neuromodulator that reduces neural activity via binding to adenosine receptors, mainly the A1 and A2a receptors. Suppressing the potency of adenosine through the antagonistic binding leads to an increase of neural activity, which may eventually cause the various positive effects of caffeine on brain function, including alleviated alertness, arousal, and attention^21–25^. These changes suggest an increase of brain information processing capacity, which can be subsequently measured with BEN. Because the antagonistic binding of caffeine is not region specific, we hypothesized that caffeine will increase BEN across a large portion of the brain. To test this hypothesis, we collected rsfMRI from a large cohort of healthy subjects before and after taking a 200 mg caffeine pill.

## Results

No significant order effects (order vs caffeine interactions) were observed on both the caffeine-induced CBF or BEN changes (two sample t-test on the post minus pre caffeine CBF or BEN changes of group A and B).

**Figure 1:**
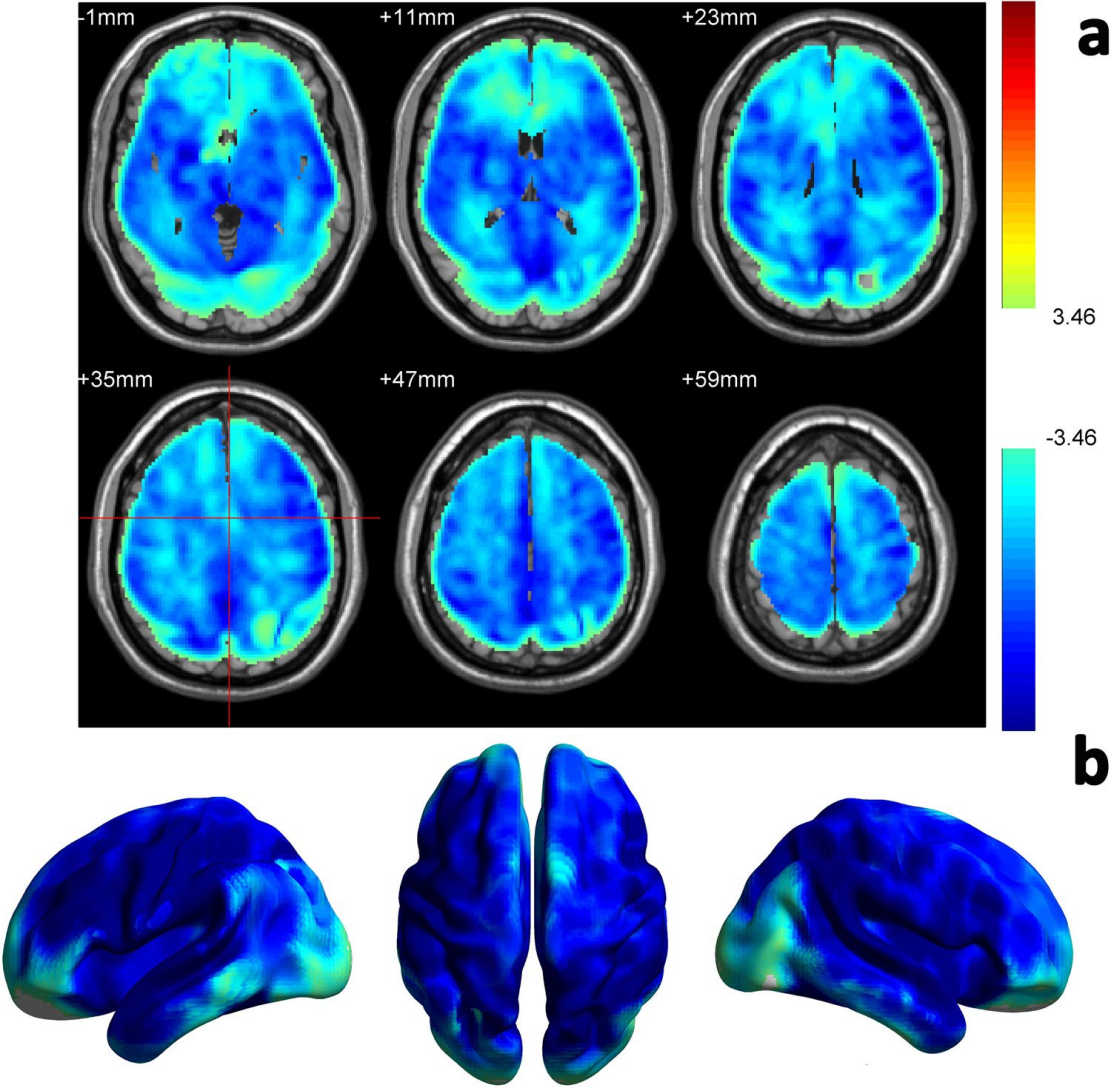
Caffeine induced whole brain CBF decrease. Paired-t test showed that compared with control condition (no caffeine), caffeine induced whole brain CBF decrease. (**a**) is the thresholded t map presented in 2D, blue means lower after caffeine ingestion, p < 0.001. (**b**) is the same result presentation in 3D.

Figure 1 shows the post-caffeine vs pre-caffeine CBF comparison results. Figure 1a shows several representative axial slices and 1B is the 3D rendering picture. Caffeine induced significant CBF reduction in the entire brain.

**Figure 2:**
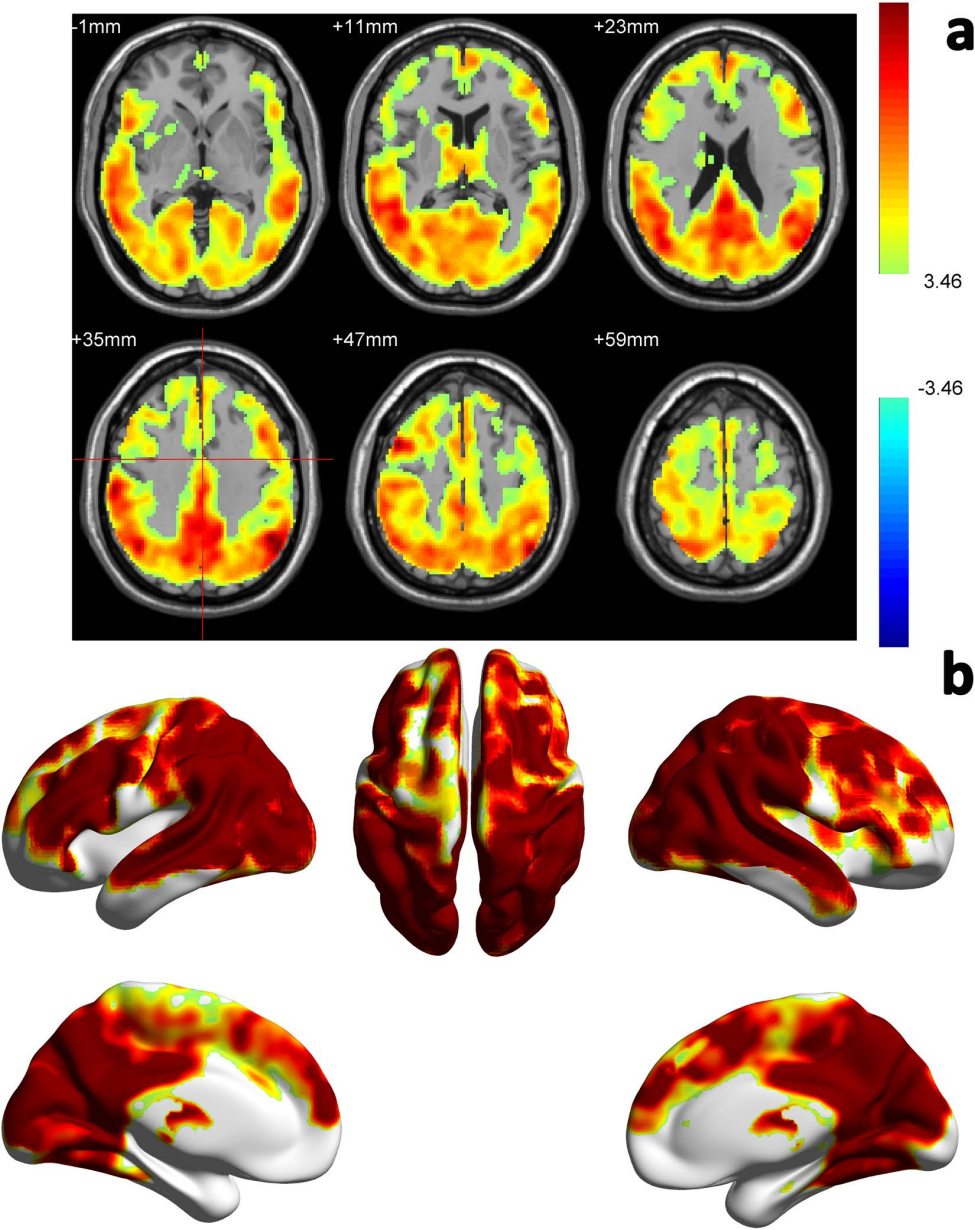
Caffeine induced BEN increase in a large portion of the cerebral cortex. Paired-t test showed that compared with control condition (no caffeine), Caffeine induced BEN increase in a large portion of the cerebral cortex with the highest increase in lateral prefrontal cortex, the DMN, visual cortex, and motor network. (**a**) is the thresholded t map presented in 2D, blue means lower after caffeine ingestion, red means higher after caffeine ingestion, p < 0.001, AlphaSim corrected (cluster size threshold is 270). (**b**) is the same result presentation in 3D.

Figure 2 is the results of caffeine induced BEN alteration shown in several axial slices (Fig. 2a) and a 3D brain (Fig. 2b). Caffeine caused BEN increase in a big portion of the cerebral cortex with the highest increase in lateral prefrontal cortex, the DMN, visual cortex, and motor network. Table 1 shows the relative decrease after caffeine ingestion in those regions. Different spatial patterns were demonstrated between the caffeine-induced CBF reduction and BEN increase.

**Table 1:** The relative increase in BEN quantity after caffeine ingestion (caffeine ingested condition minus control condition and then divided by control condition).

Brain Regions	Relative increase after caffeine ingest (%)
DMN	16.09
Visual Cortex	14.48
Lateral Prefrontal Cortex	7.70
Motor Network	8.13

**Figure 3:**
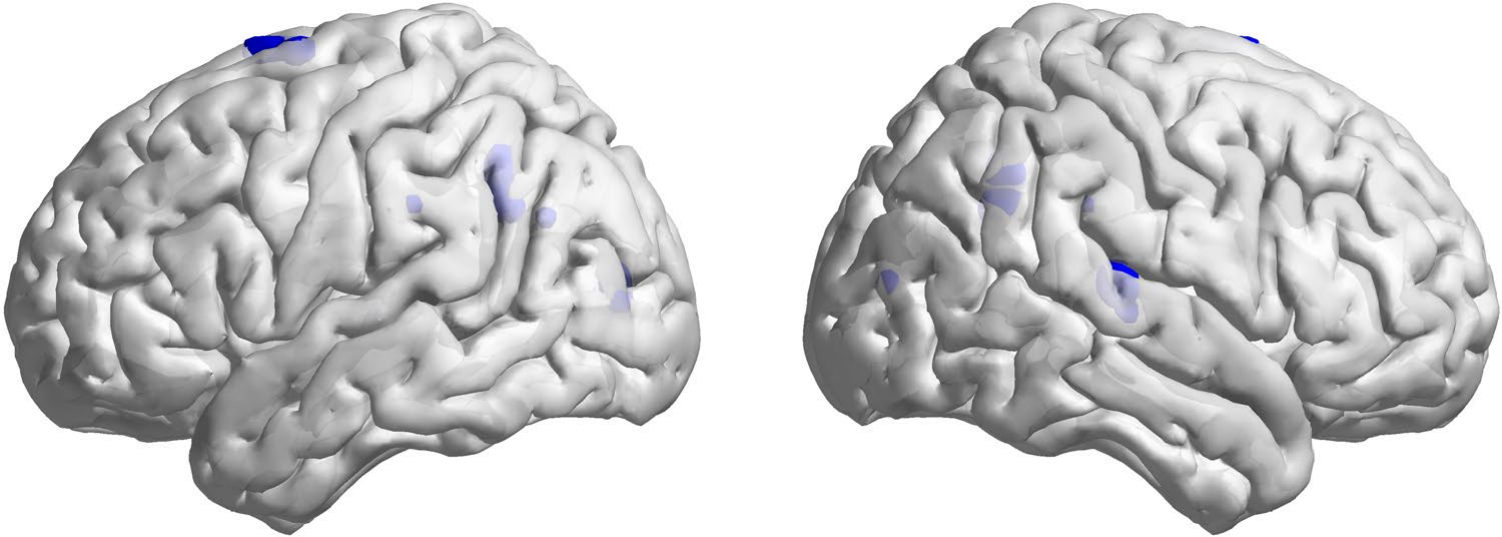
The correlation of CBF difference maps (caffeine ingested condition minus control condition) and BEN difference maps. Only tiny clusters showed moderate negative correlation. These clusters (blue spots) distributed sporadically in right superior temporal cortex, precuneus, left calcarine sulcus and supplementary motor area (r < −0.4, p < 0.02).

Figure 3 shows the voxel-wise CBF vs BEN association analysis results. Very limited brain regions showed negative correlations (−0.5 < r < −0.4) between the caffeine-induced CBF change and BEN change, including right superior temporal cortex, precuneus, left calcarine sulcus and supplementary motor area.

## Discussion

Complexity of temporal activity provides a unique window to study human brain, which is the most complex organism known to us. Temporal complexity indicates the capacity of brain for information processing and action exertions^26,27^, and has been widely assessed with entropy though these two measures don’t always align with each other - complexity doesn’t increase monotonically with entropy but rather decreases with entropy after the system reaches the maximal point of irregularity^28^. In this study, we used Sample Entropy which has been verified in^9^ and has been shown to be reproducible in normal brain and sensitivity to task performance and disease conditions^9,12–17^. In this study, we aimed to test whether SampEn-derived BEN would be changed by pharmaceutical challenge using caffeine. By comparing BEN before and after taking a small dose of caffeine in a large group (n = 60) of caffeine naïve young healthy human subjects, we identified BEN increase after caffeine ingestion, proving our hypothesis about the caffeine effects on brain activity and BEN. Increased resting BEN indicates increased resting brain activity irregularity or complexity, suggesting an increase of information processing capacity in the resting brain^29,30^.

Caffeine has both a neurovascular and neuronal effects. In this study, we observed significant CBF reduction in the entire brain after a caffeine dose, which is consistent with the general literature^29,30^. BOLD signal is mostly contributed by CBF, and large CBF change has been postulated to produce large BOLD signal variability^31^ which might be translated into higher entropy. But our data only showed correlations between BEN change and CBF change in very limited brain regions, suggesting that BEN is mostly independent of BOLD signal range (or variability). It also suggests that the observed caffeine-induced BEN increase is a neuronal effect rather than vascular one. Our findings of caffeine-induced BEN increase was partly supported by the reduced functional connectivity findings reported in^32,33^ and the increased temporal variability of rsfMRI after a caffeine does^34^ since larger temporal irregularity (increased entropy) would increase temporal variability and reduce signal correlation among different brain regions which is the base of functional connectivity. Similar to our data, those studies showed none or moderate correlation of the rsfMRI measures to the CBF changes, suggesting a non-vascular origin of the rsfMRI-derived brain measures. Nevertheless, BEN increase in part of DMN (temporal cortex, precuneus, calcarine sulcus) and motor area (SMA) still showed moderate correlations to caffeine-induced CBF reductions (larger CBF reduction corresponding to greater BEN increase), suggesting a neurovascular component of the observed BEN alterations, which is consistent with two previous caffeine rsfMRI studies^33,34^.

Caffeine-induced BEN increase varied across the brain with relatively larger BEN increase in prefrontal cortex, lateral striatum, visual cortex, and motor area. This distribution may be a result of caffeine effects on cognition: caffeine has the strongest impact on attention, vigilance, and action/motion function^35,36^ which are mainly subserved by the aforementioned brain regions. Previous EEG studies have shown that BEN increased with cognitive load^37,38^ and differed among different cognitive tasks. However, we didn’t acquire cognitive data in this study, and the above postulated BEN vs cognition relations due to caffeine can only be confirmed in future studies.

In summary, we demonstrated that caffeine increases BEN nearly in the entire cerebral cortex with more effects in DMN and sensorimotor system; the observed BEN effects are related to neuronal activity but rather to the vascular effects of caffeine.

## Methods

### Participants

This study was conducted at the Center for Cognition and Brain Disorders (CCBD) in Hangzhou Normal University. All procedures were approved by the CCBD Institutional Review Board (IRB, approval No. 20130819), and adhered to the Declaration of Helsinki. All subjects were recruited from Hangzhou Normal University and local community in Hangzhou, China. Each subject was completely informed about the procedures and the IRB approved consent form. All subjects included in this study signed the informed consent forms before any experiment procedures. Subjects were excluded if they had: abnormal structural MRI, a history of head trauma or other injury resulting in loss of consciousness lasting greater than three minutes or associated with skull fracture or inter-cranial bleeding, magnetically active objects on or within their body, any neuropsychological problems as defined by the Mini-International Neuropsychiatric Interview (MINI)^39^, or any medications that can affect CBF in the past 10 days. Additional exclusion criteria were used for the subjects recruited for the caffeine intake experiment: allergic to caffeine, drinking more than 1 cup of coffee or tea in the past week and more than 10 cups of coffee or tea in the past half year. Sixty healthy Han adults (30 males and 30 females, age: 23 ± 3 years (mean and standard deviation, age range: 19~31 years) participated in the experiment.

### Design

**Figure 4:**
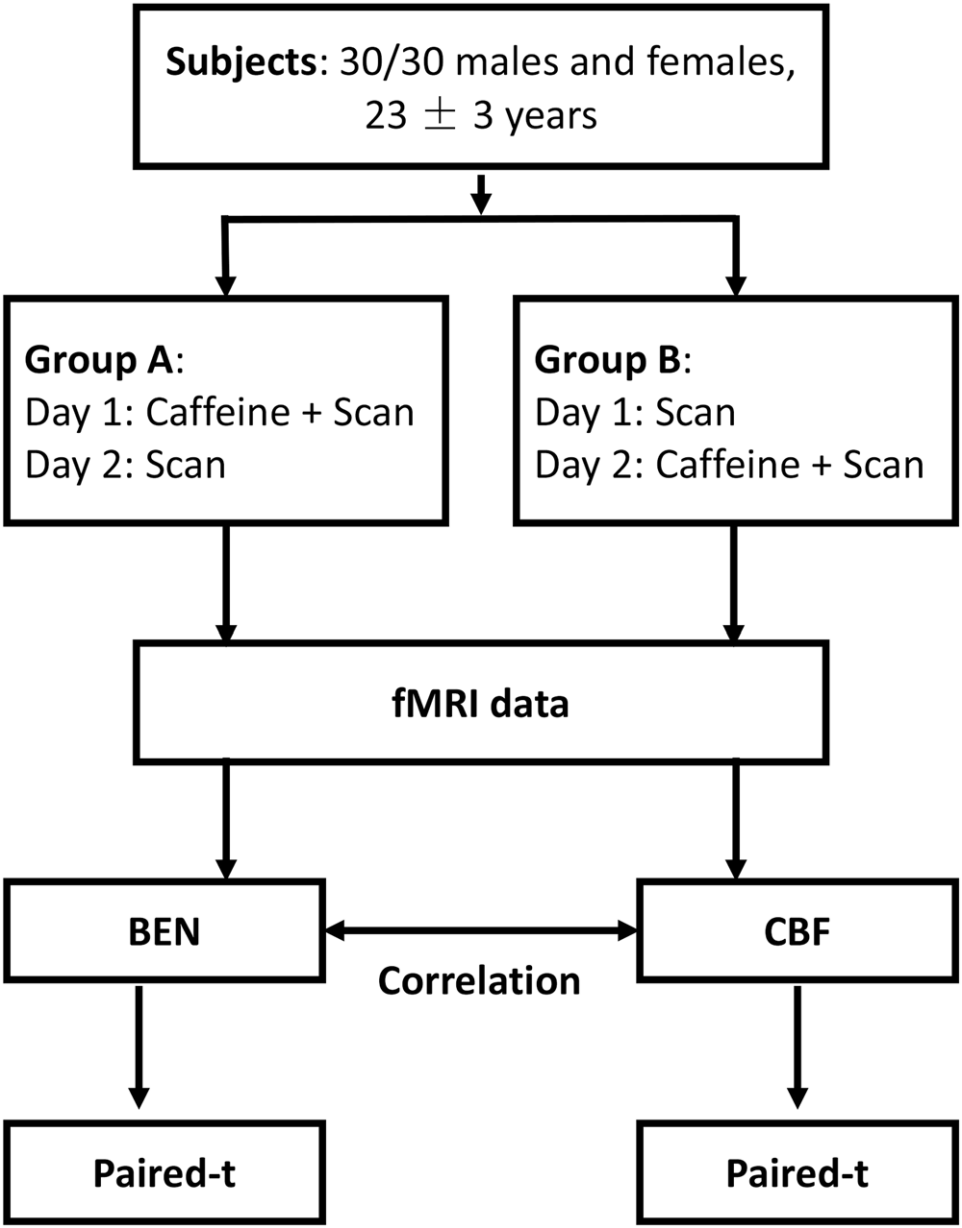
Experiment design. Resting fMRI were collected from sixty caffeine-naïve healthy subjects (30/30 males and females). Each participants participated for two days with two imaging scan session apart exactly 24 hours. For control balance, subjects are randomly assigned to two groups (each group include 15 males and 15 females), group A take caffeine pill (200 mg) with water in the first day and only the same amount of water in the second, group B take only water in the first day in turn. Paired t-test of BEN and CBF were performed and correlation of BEN and CBF is also calculated.

All participants attended two imaging sessions (one with caffeine ingestion and one without) in two days with exactly 24 hours apart during the daytime between 9 am and 5 pm. The order of sessions with or without taking caffeine prior to scan was counter-balanced with half of subjects took a caffeine pill (200 mg) in the first day. No coffee or caffeine contained beverage or food were allowed since 24 hours before the first experiment. Caffeine has a mean serum half-life of 5.7 hours^40^, and the 24 hour interval between the two scan days was chosen to allow a full digestion of caffeine if it was taken in the first scan day. For both sessions, subjects were asked to rest for the same amount of time (30 minutes) outside of the scanner room before going inside the scanner. For the caffeine ingestion session, subjects took a caffeine pill with water and waited 30 minutes before going inside the scanner for MRI; for the non-caffeine session, subjects only took the same amount of water as in the caffeine session and waited 30 minutes before going into the MR scanner. For each session, a T1-weighted high resolution structural MRI, an rsfMRI scan and an arterial spin labeling (ASL) perfusion MRI^41,42^ were acquired. The subjects were asked to stay awake and keep eyes closed while doing nothing during each scan (Fig. 4).

### Imaging Parameters

Imaging experiments were performed on a 3.0 T whole-body GE 750 MR scanner (GE, Milwaukee, US), using a standard 8-channel receive array. Structural images were acquired using a T1-weighted inversion prepared 3D spoiled gradient echo (IR-SPGR) sequence with FOV = 256 × 256 mm^2^, inversion time = 450 ms, TR/TE = 7.2/2.1 ms, 256 × 256 matrix, 176 sagittal slices with slice thickness = 1 mm. rsfMRI data were acquired with a T2^*^ weighted gradient-echo echo-planar imaging (EPI) sequence with following parameters: matrix = 64 × 64, voxel size = 3 × 3 × 3 mm^3^, TR = 2000 ms, TE = 30 ms. Thirty-seven axial slices were obtained in an interleaved order to cover the entire cerebrum and cerebellum from bottom to top. ASL MRI was acquired with a GE product fast spin echo background suppressed 3D pseudo continuous ASL (pCASL) sequence with the following parameters: TR/TE = 4690/10.9 ms, 8 shots each with a spiral readout covering 512 k-space points, 40 axial slices, voxel size = 3.389 × 3.389 × 3.4 mm^3^, number of repeat scan (NEX) = 3, labeling duration = 1500 ms, and post-labeling delay = 1525 ms, the duration of the ASL scan was 4 mins 32 secs.

### Data Processing

The rsfMRI images were preprocessed using SPM12 (http://www.fil.ion.ucl.ac.uk/spm) with the following steps: 1) discarding the first 6 images to allow signal to reach stead state; 2) the origins of both structural and raw rsfMRI images were set to be the center of the image matrix; 3) slice-timing correction; 4) motion correction; 5) registering rsfMRI to the Montreal Neurological Institute (MNI) standard brain space via each subject’s T1 weighted anatomical image; 6) temporal nuisance correction using simple regression with the residual head motions, white matter signal, and cerebrospinal fluid (CSF) signal as the co-variants; 7) linearly detrending and temporal band-pass filtering (0.01–0.08 Hz) to eliminate high-frequency noise and low-frequency drift; 8) spatial smoothing with an isotropic Gaussian kernel, full-width-at-half-maximum (FWHM) = 6-mm^3^. Each subject’s BEN map was calculated based on the preprocessed rsfMRI images with BENtbx (https://cfn.upenn.edu/~zewang/BENtbx.php) using an approximate entropy measurement, sample entropy (SampEn) with the parameters recommended in^9^; 9) individual BEN maps were spatially normalized into the Montreal Neurological Institute (MNI) space using the nonlinear transform obtained by registering the structural MR image into the MNI space using SPM12.

ASL CBF images were processed using the SPM12 based batch scripts provided in ASLtbx^43^. The following steps were included: (1) the origins of structural image, M0 map, and ASL images were set to be the center of the image matrix; (2) ASL CBF maps were calculated from the delta M and M0 maps and registered to the structural MR image for each subject; (3) CBF images were spatially normalized into the MNI using similar procedure as mentioned above; (4) CBF images were smoothed with an isotropic Gaussian filter with a FWHM = 6-mm^3^.

Paired-t tests were performed on the preprocessed BEN maps and CBF maps, respectively to characterize the caffeine induced BEN and CBF changes. Simple regression was performed at each voxel to examine the correlation of post- minus pre-caffeine BEN changes to the post- vs pre-caffeine CBF difference. Caffeine intake order effects were assessed using two-sample t-test on the post- minus pre-caffeine CBF or BEN difference of group A and B. Statistical significance was defined by p<= 0.001 at the voxel level and cluster size >= 270 (alpha < 0.5, corrected for multiple comparison using the Monte Carlo simulation based correction approach provided in AFNI (the AlphaSim tool)).
